# Universal and Lineage‐Specific Patterns in the Distribution of ECOD Domain Homology Groups Across Superkingdoms

**DOI:** 10.1002/prot.70144

**Published:** 2026-05-03

**Authors:** Rui Guo, Jimin Pei, Jing Zhang, Qian Cong, Nick V. Grishin, R. Dustin Schaeffer

**Affiliations:** ^1^ Department of Biophysics University of Texas Southwestern Medical Center Dallas Texas USA; ^2^ Eugene McDermott Center for Human Growth and Development, University of Texas Southwestern Medical Center Dallas Texas USA; ^3^ Harold C. Simmons Comprehensive Cancer Center, University of Texas Southwestern Medical Center Dallas Texas USA; ^4^ Department of Biochemistry University of Texas Southwestern Medical Center Dallas Texas USA

**Keywords:** AlphaFold proteomes, ECOD, fold architecture, protein domains, superkingdoms

## Abstract

Proteins are built from modular domains that serve as fundamental units of structure and evolution. While individual domains have been extensively cataloged, their collective distribution across the lineages of life has remained poorly resolved. Here, we use the Evolutionary Classification of Protein Domains (ECOD) to chart the occurrence of domain homology groups (H‐groups) across 44 model proteomes representing Eukaryota, Bacteria, and Archaea, in which 1.16 million domains are assigned to 3320 H‐groups. H‐groups are categorized as universal (occupying all three superkingdoms), shared between superkingdoms, or lineage‐specific. The fold architecture distributions were examined: α/β sandwiches and other mixed architectures were abundant in universal H‐groups, whereas α‐rich architectures are expanded in eukaryotic H‐groups and β‐rich folds in bacterial H‐groups. 126 (3.8%) H‐groups occur in all organisms, forming a universal structural core that supports central processes of energy conversion, metabolism, and information flow. These widely distributed folds coincide with canonical superfolds—robust, adaptable architectures repeatedly repurposed for key biochemical roles. Two superkingdom groups trace evolutionary connections between lineages: bacterial metabolic and chaperone systems inherited by eukaryotes, archaeal informational machinery conserved in eukaryotic nuclei, and ancient redox scaffolds linking bacteria and archaea. Lineage‐exclusive domains, in turn, highlight distinct adaptive strategies—regulatory and cytoskeletal innovation in eukaryotes, envelope and motility specialization in bacteria, and redox or replication refinements in archaea. Together, these data provide a quantitative, structure‐based view of protein domain evolution across the tree of life, showing that the essential architecture of life relies on a conserved set of ancient folds, while lineage‐specific diversity has largely arisen through the recombination and functional diversification of pre‐existing domains.

## 1 Introduction

Protein domains are discrete evolutionary modules within a protein molecule [1, 2, 3, 4, 5]. Each domain represents a stable and functional unit that can be recombined and repurposed across proteins and lineages [6, 7, 8, 9, 10]. Over the past decades, several databases have annotated and organized the domain universe. Structure‐based classification systems such as SCOP [11, 12], CATH [13, 14], and ECOD [15, 16] group domains by three‐dimensional architecture and evolutionary relationships, whereas sequence‐based resources like Pfam, CDD, and SUPERFAMILY classify them by conserved motifs and profiles derived from sequence alignments. These complementary hierarchies make it possible to trace how structural motifs are reused, adapted, or lost across taxa. Mapping the taxonomic distribution of domains across the tree of life offers a direct way to explore which molecular architectures form the universal core of biology and how new combinations have driven lineage‐specific innovation [17, 18, 19, 20, 21]. Previous studies have revealed that domain gains generally outnumber losses, that metabolic domains are found in proteomes across all superkingdoms, and that eukaryotic innovation largely arises from the expansion of domains involved in regulation and extracellular functions [22, 23, 24, 25, 26].

Within this context, one of the most fundamental questions concerns universality: which structural modules are indispensable to life and conserved across all lineages? Comparative genomics first approached this at the gene level through “minimal gene set” analyses, estimating roughly 250 genes as the lower bound for autonomous viability [27, 28, 29, 30]. Experiments such as the synthetic minimal cell JCVI‐syn3.0 refined this concept, showing that essential functions cluster around translation, genome maintenance, lipid and cofactor metabolism, while many indispensable genes still lack defined biochemical roles [31, 32, 33, 34]. From the structural perspective, protein fold analyses provided a complementary view. A small subset of highly populated fold families, often termed “superfolds,” recur widely across the protein universe, including the TIM barrel, Rossmann‐like, β‐propeller, and helix–turn–helix folds [35, 36, 37]. These superfolds form exceptionally stable scaffolds that accommodate diverse catalytic and binding sites, suggesting that fold reuse has been as crucial as fold invention in molecular evolution [38, 39, 40, 41]. These observations imply that tracing domain distributions across superkingdoms can reveal both the core architectures that sustain life and the lineage‐specific innovations that drive its diversification.

The advent of high‐accuracy structure prediction has transformed how protein evolution can be studied [42, 43, 44]. Earlier proteome‐level domain analyses relied primarily on sequence‐based domain assignments, as experimental structures covered only a fraction of known proteins. Such an approach had limitations, since roughly only half of the domain sequences could be confidently linked to the homolog of known structure, leaving much of the proteomic space structurally uncharacterized [45, 46]. As a result, many evolutionary relationships among distant or novel domains remained unresolved. Recent advances, particularly AlphaFold's release of proteome‐scale models and their integration into the Evolutionary Classification of Protein Domains (ECOD), have overcome this limitation [47, 48]. ECOD unifies experimentally solved and computationally predicted structures within a hierarchical framework that organizes domains by architecture, potential homology, and confirmed homology groups (A → X → H) [15, 16]. This comprehensive structural coverage enables direct, domain‐level comparisons across the entire tree of life rather than relying on sequence inference alone.

**FIGURE 1:**
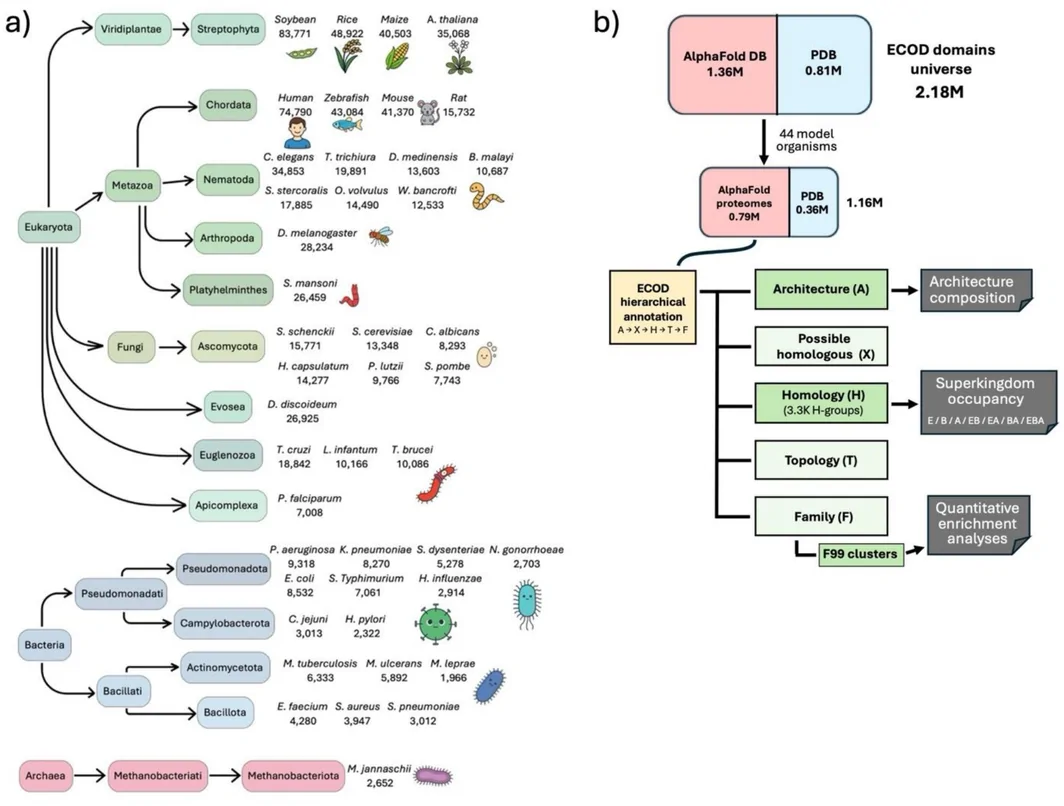
Taxonomic distribution of the 44 model organisms and overview of the ECOD analysis workflow. (a) Hierarchical taxonomic representation of the 44 model organisms analyzed in this study, organized from superkingdom to kingdom to phylum, with individual species shown under their corresponding phylum. The total number of protein sequences for each organism, clustered at 99% sequence identity, is indicated below the organism name. (b) Schematic overview of the ECOD‐based analysis workflow. Nonviral ECOD domains were partitioned by source database (AlphaFold DB and PDB), then restricted to the 44 model proteomes analyzed here. Domains were interpreted within the ECOD hierarchical annotation framework (A, architecture; X, possible homology; H, homology; T, topology; and F, family). H‐groups were used to define superkingdom occupancy categories (E, B, A, EB, EA, BA, and EBA), A‐level annotations were used to summarize architecture composition across categories, and f99 sequence clusters were used for quantitative enrichment analyses.

In this study, we analyze ECOD domains from 44 representative proteomes (Figure 1) spanning the three domains/superkingdoms Eukaryota, Bacteria, and Archaea to construct a quantitative, structure‐based census of domain evolution. By combining experimental and predicted structures, this dataset captures nearly complete proteomic coverage for key model organisms, allowing us to identify universal, shared, and lineage‐specific structural groups. These data provide a foundation for examining how life's molecular diversity arises from the reorganization and reuse of a limited set of ancient structural architectures.

## 2 Results and Discussion

### 2.1 Proteome Dataset (44 Datasets) and Overall Distribution of Domain Groups Across Superkingdoms

We analyzed ECOD domains from 44 representative model organisms spanning 28 eukaryotes, 15 bacteria, and 1 archaeon ( *Methanocaldococcus jannaschii* ) (Figure 1; full taxonomy in Table S1). The eukaryotic set covers major kingdom lineages, including plants (Streptophyta), animals (Chordata, Nematoda, Platyhelminthes, Arthropoda), fungi (Ascomycota), and several protists (Evosea, Euglenozoa, and Apicomplexa). Bacteria are represented by major phyla such as Pseudomonadota, Bacillota, Actinomycetota, and Campylobacterota. These 44 species provide a compact but phylogenetically balanced view of the tree of life (Figure 1a), encompassing both multicellular and unicellular organisms with diverse metabolic and ecological strategies. A total of 1.16 million ECOD domains were identified across these proteomes (Figure 1b). To prevent inflation from near‐identical sequences and uneven structural sampling, all domains were clustered at 99% sequence identity using CD‐HIT, yielding 671 582 nonredundant sequence clusters (hereafter referred to as f99 clusters). ECOD provides predefined sequence clustering levels at 99%, 70%, and 40% identity; we selected the 99% threshold because it collapses only near‐identical sequences (e.g., strain variants or highly similar homologs) while preserving distinctions between more diverged domains. This choice ensures that redundancy is reduced primarily within closely related organisms, typically within the same superkingdom, while avoiding the merging of more divergent sequences that may span multiple superkingdoms. In this framework, multiple nearly identical domain sequences contribute a single count, providing a conservative and standardized estimate of domain representation across organisms. Unless otherwise noted, quantitative analyses in this study, including superkingdom occupancy and enrichment calculations, are based on f99 clusters rather than raw domain counts. ECOD incorporates the full 48 AlphaFold proteomes set; four (*Cladophialophora carrionii*, *Fonsecaea pedrosoi*, *Madurella mycetomatis*, and *Nocardia brasiliensis* ) were excluded because their domains have not yet been assigned to f99 clusters in the current release. Of the 1.16 million domains analyzed, 68.5% originated from AlphaFold‐predicted models and 31.5% were derived from experimentally determined PDB structures. However, AlphaFold entries account for more than 95% of all unique sequences, whereas PDB structures cover only about 8%, highlighting the strong redundancy and sampling bias present in experimental structural datasets. This bias is amplified by the fact that many experimentally studied proteins are represented by numerous near‐identical variants, including constructs carrying one or a few engineered mutations used in structure–function analyses. The inclusion of full AlphaFold proteomes enables domain‐level analyses that were previously infeasible using PDB data alone, providing near‐exhaustive structural representation across the three superkingdoms.

**FIGURE 2:**
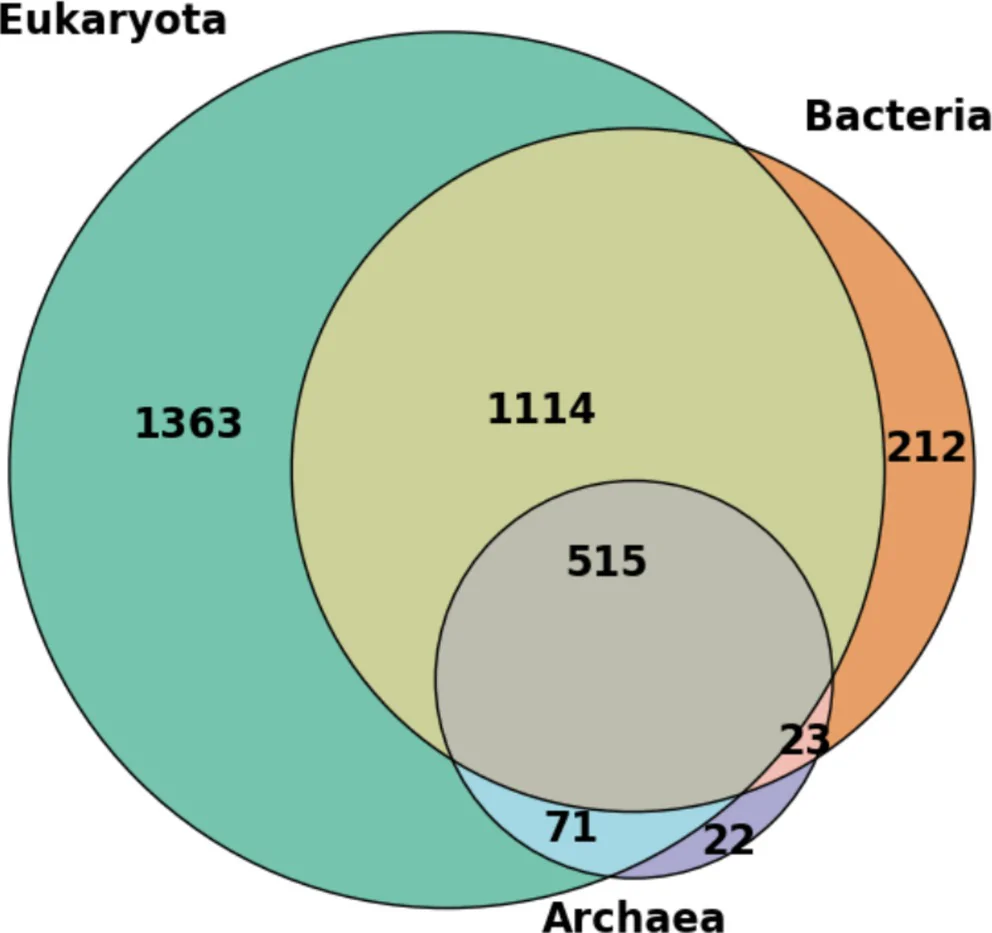
Distribution of ECOD H‐groups across superkingdoms. Venn diagram showing the overlap of 3320 ECOD H‐groups among Eukaryota (E), Bacteria (B), and Archaea (A) across 44 model proteomes. Numbers in each region denote the count of H‐groups present in that combination of lineages.

Within the global ECOD database, 2 176 265 domains are grouped into 1 039 314 f99 clusters across 11 479 organisms. The 44 model organisms analyzed here contribute 1 159 312 domains (53.3%) and 671 582 f99 clusters (64.6%), highlighting their substantial coverage despite representing fewer than 0.1% of all species in ECOD. These proteomes encompass 3320 of 3614 H‐groups (92%) in the current database. The 294 H‐groups absent from this reduced dataset are all small (≤ 21 clusters each); examples among the biggest absent H‐groups are the subtilisin inhibitor (H‐group ID 841.1, with 21 f99 clusters), the N‐terminal domain of the GerK3 germinant receptor (7506.1, 16 f99 clusters), and the variable surface antigen VlsE (624.1, 15 clusters). The top 20 H‐groups absent from the model organism set are listed in Table S2. These folds likely represent narrow, lineage‐specific functions not captured among the 44 model proteomes rather than major structural omissions. Across these proteomes, the 3320 ECOD H‐groups were categorized by their taxonomic presence within the three superkingdoms: Eukaryota (E), Bacteria (B), and Archaea (A). The distribution pattern is shown in a Venn diagram in Figure 2. A total of 515 H‐groups are shared by all three lineages (EBA), 1207 occur in exactly two (EB = 1114; EA = 71; and BA = 23), and 1597 are restricted to a single superkingdom (E = 1363; B = 212; and A = 22).

### 2.2 Architecture Distribution Across Superkingdom Categories

In the ECOD hierarchy, domains are annotated at multiple structural levels: A (architecture) → X (possible homology) → H (homology) → T (topology) → F (family). Architecture (A‐level) distributions of the H‐groups were examined to investigate large‐scale structural trends. Twenty architectures were represented in total, encompassing five primarily α‐type categories, eight mixed α + β or α/β classes, five β‐type categories, and two containing irregular or extended topologies. For each superkingdom‐membership category (EBA, EB, EA, BA, E, B, and A), the fraction of H‐groups belonging to each architecture was calculated.

**FIGURE 3:**
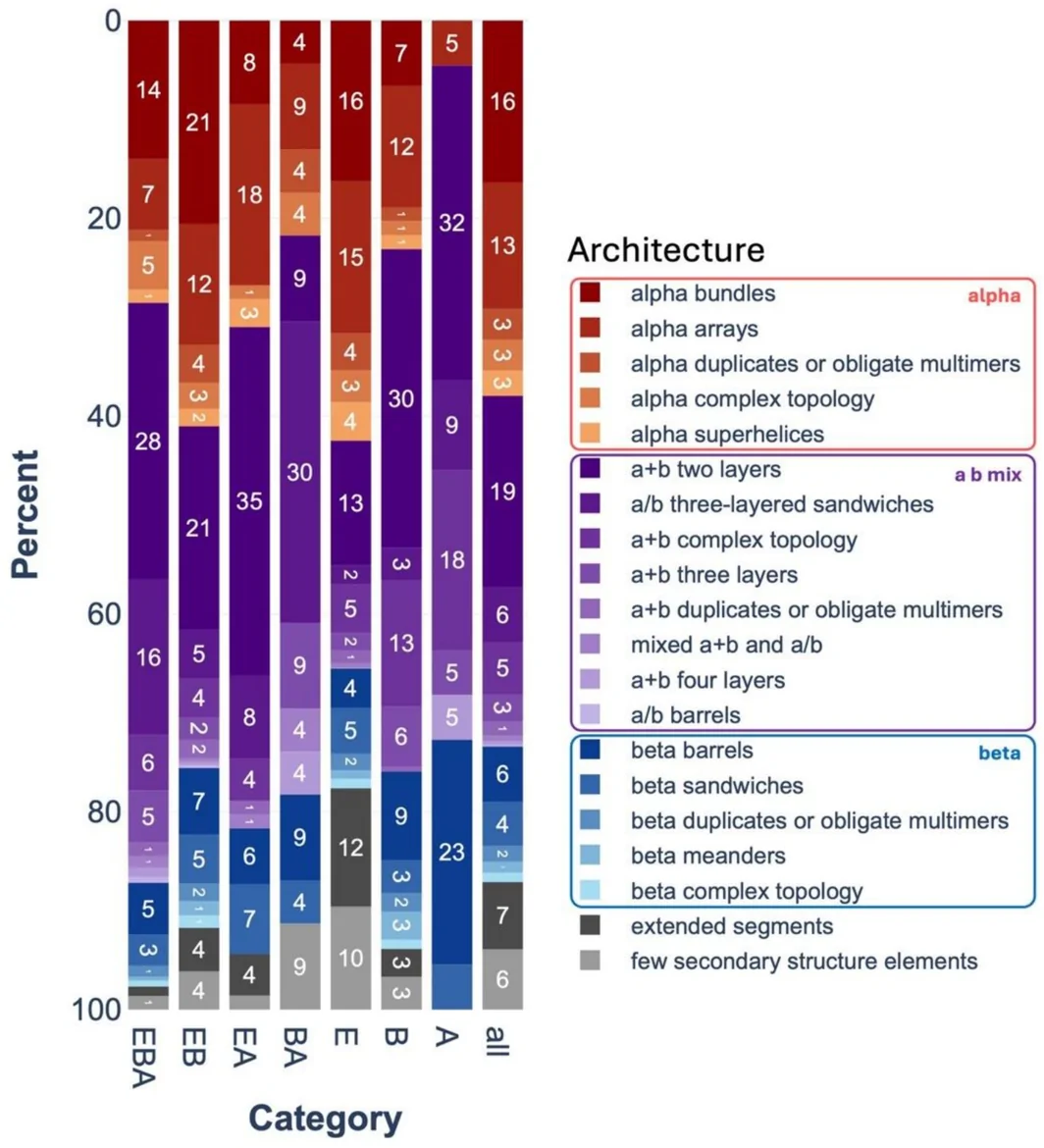
Architecture distribution of ECOD H‐groups by superkingdom category. Each domain homology group (H‐group) from the 44‐proteome dataset was assigned to its corresponding A‐level architecture in the ECOD hierarchy (A → X → H → T → F). For each superkingdom‐membership class: Universal (EBA), shared between two superkingdoms (EB, EA, and BA), or exclusive to one (E, B, and A), the proportion of H‐groups belonging to each architecture was calculated and plotted as stacked bars. Each H‐group contributes one count to its assigned architecture. Color themes highlight secondary structure groups: Mainly α in the red theme, mainly β in the blue theme, and α + β α / β in the purple theme. The last bar “all” shows the background distribution of all 3320 H‐groups combined. Percentage numbers are labeled when an architecture constitutes more than 1% of the total for that category.

Among all 3320 H‐groups, the α/β three‐layered sandwich architecture is the most populated (19%), followed by α‐bundles (16%) and α + β two‐layered structures (13%), as shown in Figure 3. While the general pattern is similar among various taxonomic categories, notable shifts are observed. Eukaryote‐only domains are the most α‐rich, with α architectures accounting for ~40% of their total, reflecting the abundance of helical scaffolds in regulatory and structural proteins. Bacteria‐only domains show an intermediate profile (~22% α) with increased β‐sandwich and β‐barrel content typical of outer‐membrane and envelope proteins. Archaea‐only domains (with only 22 H‐groups) are enriched in α + β and β architectures, consistent with the predominance of redox and cofactor enzymes in this lineage. Among shared sets, α/β three‐layered sandwiches remain consistently overrepresented in EBA, EA, and BA groups, indicating that mixed α/β scaffolds are important across superkingdoms. Even at the coarse architecture level, the structural composition of domain repertoires varies systematically with evolutionary breadth. Since the BA and A categories contain relatively few H‐groups (23 and 22, respectively), their percentages should be interpreted qualitatively rather than quantitatively. Nonetheless, the architecture‐level census highlights that global fold usage is not uniform: lineages and lineage combinations differ in their reliance on α, β, or mixed α + β / α/β architectures, reflecting distinct structural strategies underlying metabolic, informational, and regulatory evolution.

**FIGURE 4:**
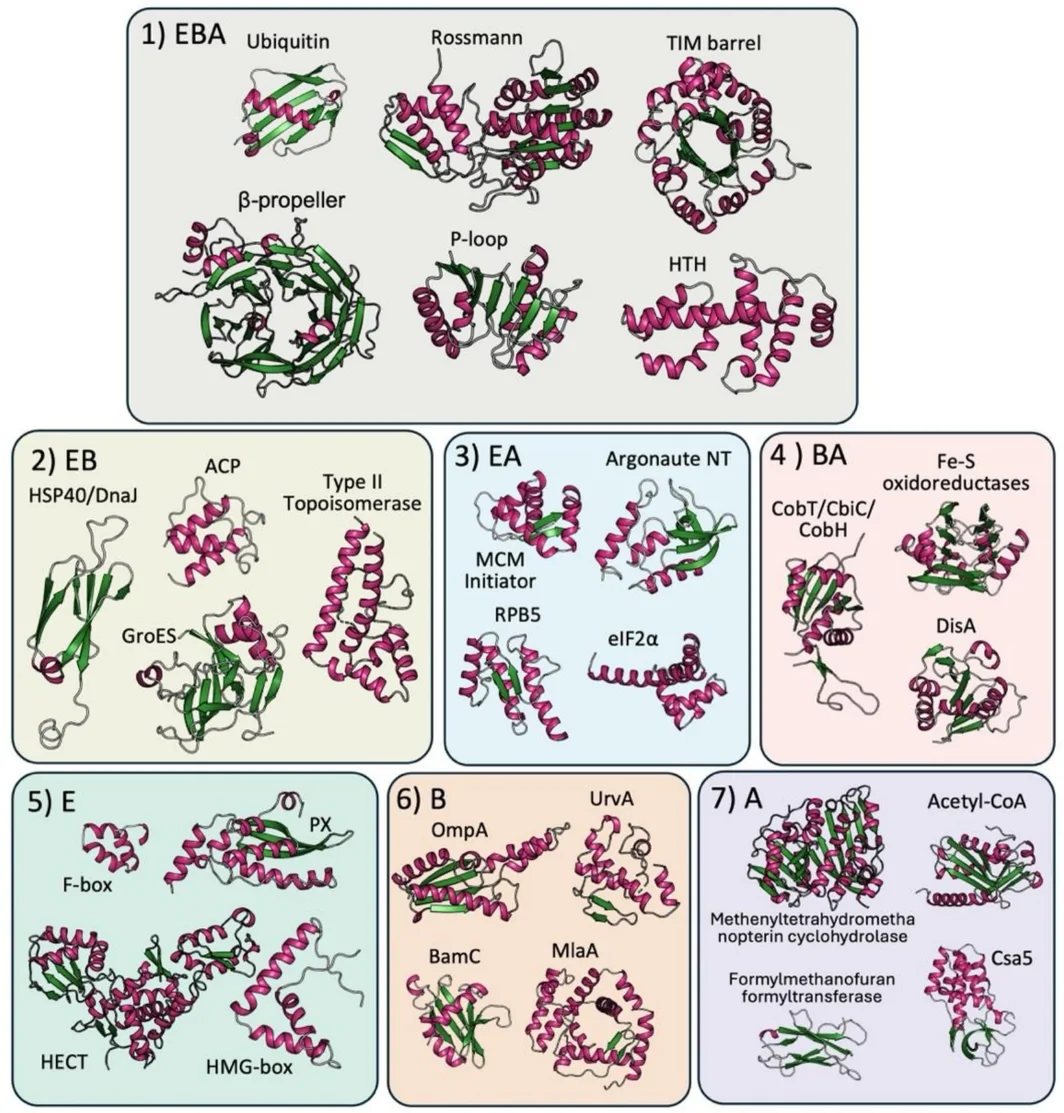
Representative ECOD H‐groups across superkingdom categories. Structure representations of selected domains from each taxonomical group. The seven categories (EBA, EB, EA, BA, E, B, and A) correspond to the regions of the Venn diagram in Figure 2, representing a hierarchy from universally conserved domains (EBA), to domains shared between two superkingdoms (EB, EA, and BA), to lineage‐specific domains restricted to a single superkingdom (E, B, and A). Representative H‐groups are selected from Table 1. Structures are rendered in cartoon representation and colored by secondary structure (α‐helices = pink, β‐strands = green).

Representative structure examples of H‐groups from each superkingdom category are illustrated in Figure 4. These structures highlight the diversity of domain architectures found in universal, shared, and lineage‐specific sets. These groups are listed in Table 1 and are discussed in greater detail in the following sections.

**TABLE 1:** Representative ECOD H‐groups and predominant functional themes across superkingdom categories.

Superkingdom category	Functional theme	Representative H‐groups
EBA (Universal)	Energy metabolism and nucleotide/cofactor handling	P‐loop NTPase (2004.1); Rossmann (2003.1); TIM barrel (2002.1); HAD (2006.1); ThDP‐binding (7574.1); and PLP‐transferase (7577.1)
	DNA replication and repair	RNase H‐like (2484.1); H2TH (102.2); HD‐domain/PDEase (131.1); UDG (7569.1); GIY–YIG endonuclease (821.1); and dUTPase (70.2)
	Transcription and RNA processing	HTH (101.1); RIFT (1.1); KH domain (327.11); and ββα “swiveling” RNAP domain (2487.1)
	Translation machinery	Class II aaRS (314.1); aaRS anticodon‐binding (7502.1); EF‐G C‐term (304.24); and IF2/eIF5B (7526.1)
	Proteostasis and chaperones	Hsp90 ATPase (225.1); ClpP/crotonase (2486.1); FtsH (4070.1); Thioredoxin‐like (2485.1); and FKBP (284.1)
	Redox and metal handling	4Fe–4S ferredoxin (205.1); Rubredoxin (375.1); ferritin/heme oxygenase (150.1); zincin metalloprotease (2498.1); and metallo‐phosphatase (246.2)
	Membrane transport and homeostasis	MFS transporter (5050.1); Drug/Metabolite transporter (5059.1); CPA antiporter (3236.1); UraA (3226.1); and ABC exporter TMD (1075.1)
EB (Eukaryota + Bacteria)	Lipid metabolism and membrane biogenesis	Acyl‐carrier protein (132.1) and α/β‐Hydrolase (7579.1)
	Chaperone systems	GroES (236.1) and Hsp40/DnaJ (67.1)
	DNA topology	Type II topoisomerase (4014.1, 4016.1)
EA (Eukaryota + Archaea)	Replication initiation	MCM (3003.1) and GINS (4163.1)
	Transcription and translation factors	RPB5‐like RNAP (840.1); eIF2α (102.3, 304.19); and Argonaute N‐term (304.112)
BA (Bacteria + Archaea)	Cofactor biosynthesis (B12)	CobT/CbiC/CobH (7553.1, 2007.7)
	Anaerobic redox metabolism and stress signaling	Fe–S oxidoreductases (806.1, 169.1, 3858.1); DisA (4279.1); and STT3/PglB β‐barrel (3111.1)
Eukaryota only	Ubiquitin signaling and protein turnover	F‐box (145.1) and HECT E3 ligase (261.1)
	Chromatin and transcriptional regulation	Bromodomain (3525.1) and HMG‐box (190.1)
	Cytoskeleton and morphogenesis	FH2 (626.1); Gelsolin (224.1); and PX domain (277.1)
Bacteria only	Cell envelope and membrane assembly	OmpA (301.3); Wza (6016.1); and MlaA (3466.1)
	Motility and chemotaxis	FlgE (4167.1); CheW (2.1); and FliD (601.5)
	DNA repair and transcription regulation	UvrA (3326.1); σ^54^ (1004.1); and PBP‐2x (294.2)
Archaea only	Methanogenesis and archaeal metabolism	Formylmethanofuran transferase (304.21); Methenyltetrahydromethanopterin cyclohydrolase (869.1); and Acetyl‐CoA synthase (4223.1, 4971.1)
	Archaeal replication and CRISPR defense	DNA polymerase D (3349.1) and Csa5 (3572.1)

*Note:* Representative H‐groups from each superkingdom occupancy class (EBA, EB, EA, BA, E, B, and A) are organized according to broad functional themes discussed in the main text. H‐group IDs correspond to ECOD homology group identifiers. The table summarizes selected examples for conceptual illustration; complete quantitative listings of all H‐groups within each category are provided in Tables S3–S9. Functional themes reflect predominant roles reported for canonical members of each structural family and are supported by representative literature cited in the corresponding Section 2.

### 2.3 Universal Domain Set and Functional Architecture (EBA)

Among all 3320 H‐groups, 515 (15.5%) are shared by all three superkingdoms—Eukaryota, Bacteria, and Archaea (Figure 2). These EBA domains define the structural “common core” of life, representing folds that predate the deepest evolutionary divergences. Within this universal subset, 126 H‐groups occur in each of the 44 species analyzed (Table 1, full list in Table S3), forming the most conserved layer of protein evolution—a compact toolkit of structural solutions that collectively sustain cellular life. Note that *M. jannaschii* , the sole archaeal representative, shares 515 of its 631 H‐groups' repertoire with Bacteria and Eukaryota, highlighting its retention of ancient, LUCA (last universal common ancestor)‐like biochemistry.

Functionally, the universal H‐groups cluster around two central biochemical imperatives: energy conversion and information maintenance, with additional conserved roles in proteostasis, redox chemistry, and membrane homeostasis (Table 1). Energy‐ and cofactor‐handling scaffolds, including P‐loop NTPases, Rossmann‐like domains, TIM barrels, HAD‐like phosphatase folds, and ThDP/PLP‐dependent enzymes, are predominantly associated with metabolism and nucleotide/cofactor processing [49, 50, 51, 52, 53, 54]. Information‐processing functions are represented by H‐groups involved in DNA replication and repair, transcription and RNA processing, and translation machinery, including RNase H‐like domains, helix–turn–helix proteins, KH‐domain proteins, RIFT‐related folds, aminoacyl‐tRNA synthetase‐associated domains, and translation factors such as EF‐G and IF2/eIF5B [55, 56, 57, 58, 59, 60, 61]. Additional universal themes include proteostasis and chaperone systems, represented by Hsp90 ATPase, ClpP/crotonase, FtsH, thioredoxin‐like, and FKBP‐like folds [62, 63, 64, 65]; redox and metal‐handling functions, represented by ferredoxins, rubredoxins, ferritin/heme oxygenase‐like proteins, zincin metalloproteases, and metallo‐phosphatases [65, 66, 67, 68]; and membrane transport and homeostasis, represented by MFS transporters, drug/metabolite transporters, CPA antiporters, UraA, and ABC exporter transmembrane domains [69, 70].

These same processes dominate minimal gene sets and synthetic minimal cells [27, 28, 31], confirming that universality at the structural level mirrors essentiality at the functional level. Even when gene sequences diverge through non‐orthologous replacement, the underlying fold architectures persist, emphasizing that evolution conserves structures, not specific genes. Universal folds are not only ancient but also expansive. Their average f99 cluster count (~2500) is more than tenfold higher than the global mean (~200), indicating that highly conserved scaffolds also serve as the most sequence‐diverse and evolvable. Many correspond to classic “superfolds” such as the Rossmann, TIM barrel, β‐propeller, and SH3 architectures known for exceptional stability and catalytic flexibility. The widespread conservation, structural robustness, and functional versatility of these universal folds suggest that they may serve as favorable scaffolds for protein engineering and heterologous expression, as their stability and adaptability have enabled their successful reuse across diverse cellular environments.

**FIGURE 5:**
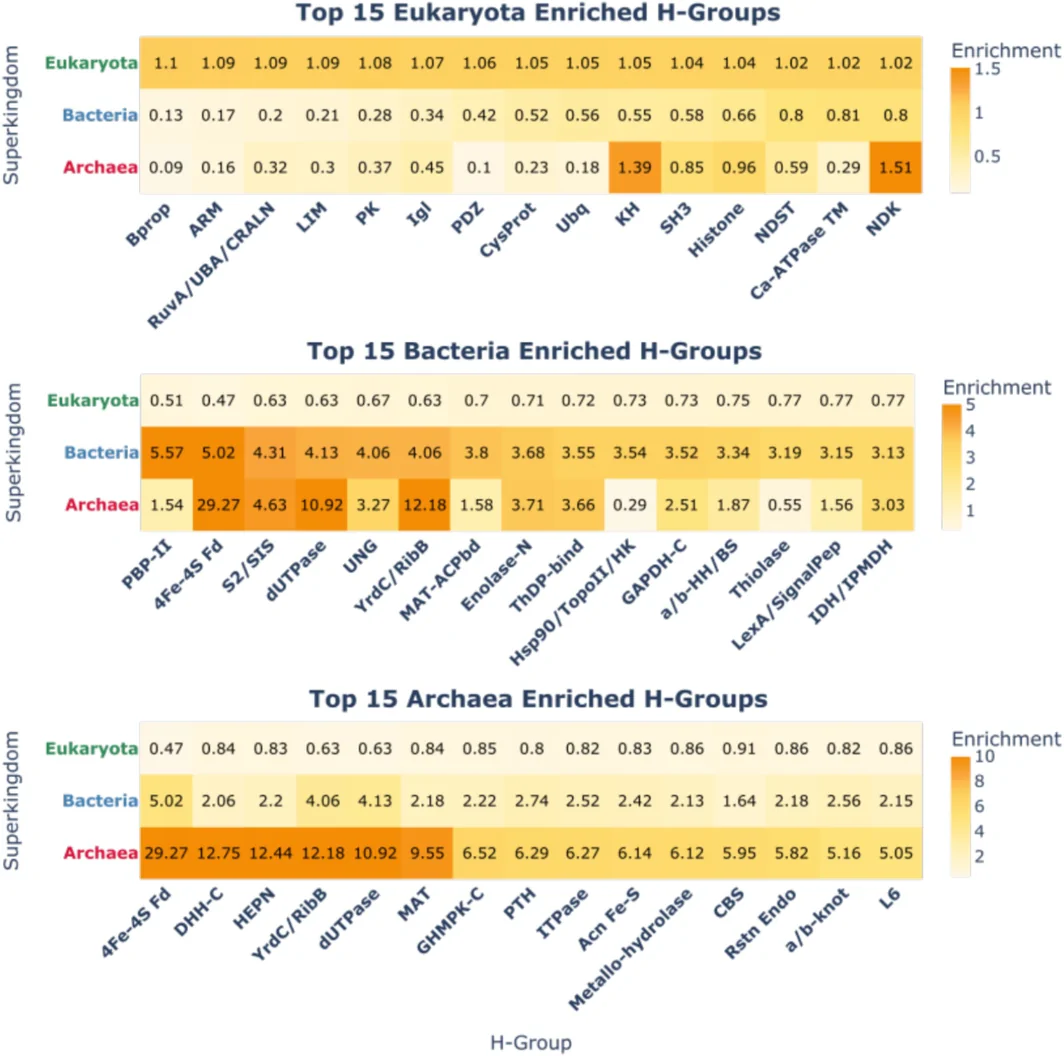
Lineage‐specific enrichment of universal H‐groups. Heatmaps showing superkingdom enrichment ratios for the 126 H‐groups present in all 44 proteomes. Each enrichment score represents the observed fraction of f99 clusters belonging to a given lineage (Eukaryota, Bacteria, or Archaea) divided by that lineage's global background frequency (E = 0.9009, B = 0.0958, and A = 0.00339). Values greater than 1 indicate overrepresentation relative to the overall dataset. The top 15 H‐groups most enriched in each superkingdom are displayed. H‐group names are shortened; full names are listed in Table S10.

Despite their shared presence, the 126 universally conserved H‐groups show measurable lineage biases in how strongly they are represented within each domain of life. Superkingdom enrichment scores for each H‐group were calculated to quantify these trends (Figure 5), defined as the ratio between its observed frequency of f99 clusters in a given lineage and the background frequency of clusters across all domains. Scores above 1 therefore indicate folds that occur more often in that lineage than expected by chance. The top 15 H‐groups with the highest enrichment in eukaryotes, bacteria, and archaea are shown in Figure 5 as separate heatmaps. Eukaryotes modestly amplify repeat‐based scaffolds like β‐propellers, ARM repeats, and other interaction domains supporting regulatory complexity. Bacteria favor metabolic and envelope‐related folds such as ferredoxins, enolases, and periplasmic‐binding proteins. Archaea concentrate on Fe–S redox enzymes, SAM‐dependent cofactors, and RNA‐processing modules characteristic of anaerobic metabolism.

### 2.4 Domains Shared by Two Superkingdoms (EB, EA, and BA)

Between the universal core of life and the domains restricted to individual lineages lies an intermediate set of 1207 H‐groups shared by exactly two of the three superkingdoms (Tables S4–S6). These “bridge” domains offer a window into the evolutionary transitions that shaped the modern proteome, highlighting how inheritance, symbiosis, and divergence collectively built the mosaic of present‐day protein architectures. A large portion (1114 H‐groups) of these shared domains connects eukaryotes and bacteria (EB), reflecting the bacterial contribution to eukaryotic metabolic capacity, among which 24 H‐groups exist in all Eukaryota and Bacteria organisms (Table S4). As summarized in Table 1, EB groups are enriched in functions related to lipid metabolism and membrane biogenesis, chaperone systems, and DNA topology. Representative examples include acyl‐carrier proteins and α/β‐hydrolase folds associated with lipid metabolism, GroES and Hsp40/DnaJ chaperones involved in protein folding, and type II topoisomerases involved in DNA topology control [71, 72, 73, 74, 75]. Their absence in archaea parallels fundamental differences in archaeal membrane composition and proteostasis systems. These features suggest that many EB domains trace back to the bacterial partners of endosymbiotic events, forming the metabolic and chaperone systems that eukaryotes inherited through mitochondrial and plastid origins.

A total of 71 H‐groups link eukaryotes and archaea (EA), in which 23 H‐groups exist in all EA organisms (Table S5). EA groups are characterized primarily by information‐processing functions, especially replication initiation, transcription, and translation‐related factors, as reflected in Table 1. Representative folds include MCM helicases and GINS complexes involved in DNA replication, together with RNA polymerase‐associated and eIF2‐related domains linked to transcription and translation [76, 77, 78]. This distribution connects directly to the on‐going debates in molecular evolution: whether life is best represented by three primary domains or by a two‐domain “eocyte” tree, in which eukaryotes branch from within the archaeal radiation rather than forming a separate lineage [79, 80, 81, 82, 83, 84, 85]. The traditional three‐domain framework, established from ribosomal RNA phylogenies, interprets the superkingdoms as distinct, coequal lineages diverging from a universal ancestor. In contrast, more recent phylogenomic analyses using conserved proteins and improved evolutionary models increasingly support a two‐domain topology, placing eukaryotes as descendants of an archaeal host that incorporated an alphaproteobacterial endosymbiont—the ancestral mitochondrion [86, 87, 88, 89, 90]. Our EA‐restricted domains are consistent with this latter view: their concentration in replication, transcription, and translation functions mirrors the informational gene set shared between archaea and the eukaryotic nucleus, whereas bacterial‐type metabolic and chaperone systems dominate the EB category. These patterns reinforce a chimeric origin of eukaryotes, in which informational architectures were inherited from archaeal ancestors and metabolic scaffolds from bacterial partners, aligning with the two‐domain tree while remaining compatible with a deep archaeal–bacterial divergence at life's root.

Only 23 H‐groups are shared exclusively between bacteria and archaea (Table S6), forming the smallest of the two superkingdom categories. Despite their limited number, these BA domains are functionally cohesive and point toward ancient prokaryotic chemistries that predate the rise of eukaryotes. As summarized in Table 1, the dominant themes in this group are cofactor biosynthesis, anaerobic redox metabolism, and stress‐associated systems. Representative folds include CobT/CbiC/CobH enzymes involved in cobalamin (B12) biosynthesis, several Fe–S oxidoreductase families that mediate anaerobic redox reactions, the DisA checkpoint/signaling domain, and the STT3/PglB β‐barrel associated with N‐glycosylation‐related systems [91, 92]. Their confinement to bacteria and archaea likely reflects metabolic pathways and envelope‐associated systems tied to early prokaryotic physiology (particularly anaerobic cofactors, redox networks, and stress responses) that were either lost, compartmentalized, or replaced during eukaryotic evolution. As such, BA domains mark a vestigial layer of prokaryotic biochemistry that persists across both microbial superkingdoms but played little role in the emergence of eukaryotic cellular organization.

These two superkingdom domain groups illustrate an asymmetric pattern in the evolution of cellular systems. EB domains preserve bacterial metabolic legacies, EA domains maintain archaeal informational continuity, and BA domains reflect core prokaryotic chemistries likely inherited from early ancestors. This layered distribution suggests that eukaryotes emerged from the integration of archaeal information systems with bacterial metabolic systems, a composite history that remains encoded in the structural repertoire of contemporary protein folds. The intermediate distribution of these two superkingdom domains may also reflect structural and functional compatibility across lineages, suggesting that such folds could be more readily adapted or transferred between biological systems, including in heterologous or synthetic contexts.

### 2.5 Lineage‐Specific Domain Groups (E, B, and A)

A total of 1597 H‐groups are restricted to a single superkingdom (Table 1, Tables S7–S9), representing the most recent and specialized layers of domain evolution. Of these, 1363 are unique to eukaryotes, 212 to bacteria, and 22 to archaea. The strong asymmetry reflects both biological and sampling factors: the eukaryotic set spans highly diversified lineages (plants, animals, fungi, and protists), each characterized by complex cellular organization and extensive domain recombination.

Among the 1363 H‐groups found exclusively in eukaryotes, a smaller conserved subset of 63 occurs in all 28 sampled organisms (Table S7). As summarized in Table 1, these eukaryote‐specific folds are dominated by three broad functional themes: ubiquitin signaling and protein turnover, chromatin and transcriptional regulation, and cytoskeleton‐associated morphogenesis. Representative examples include F‐box and HECT E3 ligase domains involved in ubiquitin‐mediated regulation, bromodomains and HMG‐box folds associated with chromatin organization and transcriptional control, and FH2, gelsolin, and PX‐domain proteins involved in cytoskeletal dynamics and membrane‐associated trafficking [93, 94, 95, 96, 97]. Their broad distribution suggests that the shared foundation of eukaryotic complexity lies not in new enzymatic chemistries, but in regulatory integration built from pre‐existing structural motifs. The remaining, more narrowly distributed eukaryote‐exclusive domains might encode lineage‐specific metabolic and ecological adaptations but were not examined here.

Bacterial‐exclusive domains (212 H‐groups, Table S8), by contrast, are enriched in structures that maintain cell integrity and environmental responsiveness. As summarized in Table 1, the major functional themes in this set include cell envelope and membrane assembly, motility and chemotaxis, and DNA repair and transcriptional regulation. Outer‐membrane‐associated folds such as OmpA, Wza, and MlaA, together with peptidoglycan‐related enzymes such as PBP‐2x, contribute to the architecture and resilience of the bacterial cell envelope [98, 99]. Motility and chemotaxis domains, including FlgE, FliD, and CheW, support dynamic responses to external stimuli [100], while UvrA‐ and σ^54^‐related folds contribute to bacterial‐specific DNA repair and transcriptional regulation [101, 102]. These folds reflect optimization for cellular autonomy within compact genomes, emphasizing structural efficiency and rapid adaptability.

Archaea‐exclusive domains are few (22 H‐groups, Table S9) but functionally cohesive. As reflected in Table 1, they are concentrated in two major themes: methanogenesis‐related metabolism and archaeal‐specific replication and defense systems. Representative examples include formylmethanofuran transferase, methenyltetrahydromethanopterin cyclohydrolase, and acetyl‐CoA synthase‐associated folds involved in methanogenic and redox metabolism [103], together with DNA polymerase D and the CRISPR‐associated Csa5 fold that mark archaeal‐specific replication and antiviral defense [104, 105]. Their limited number likely reflects both the smaller representation of archaeal proteomes and the evolutionary conservatism of this lineage, which tends to refine ancestral mechanisms rather than introduce new structural architectures.

Across all superkingdoms, these lineage‐specific sets reveal contrasting evolutionary strategies. Eukaryotes expand functional diversity primarily through domain recombination and regulatory specialization, bacteria refine robustness and adaptability through structural optimization, and archaea maintain continuity in ancient metabolic systems. The conserved subset of eukaryote‐specific folds, dominated by signaling, trafficking, and chromatin‐regulating architectures, suggests that the shared foundation of eukaryotic complexity arises chiefly from the diversification of regulatory systems built on pre‐existing structural motifs. Broader eukaryotic innovations beyond this conserved layer likely involve additional lineage‐specific chemistries not captured in the current analysis. These lineage‐specific domains may encode structural features tailored to particular cellular environments, potentially limiting their portability across systems and highlighting the importance of evolutionary context when considering protein function or expression in heterologous hosts.

## 3 Conclusions

Our analysis of 3320 ECOD domain homology groups across 44 representative proteomes provides a structure‐resolved view of how life's molecular diversity is organized across the three superkingdoms. Fold distributions differ at the architecture level: mixed α/β architectures dominate the universal core, α‐rich domains are expanded in eukaryotic regulatory systems, and β‐rich architectures are prevalent in bacterial envelopes. These structural preferences reflect lineage‐specific functional demands rather than random variation. A small set of 126 universal H‐groups found in every single organism is involved in essential biochemical processes, which include canonical superfolds such as the Rossmann, TIM barrel, β‐propeller, SH3/PDZ, and P‐loop. Two superkingdom groups reveal evolutionary bridges: bacterial metabolic and chaperone systems retained by eukaryotes; archaeal informational machinery mirrored in the eukaryotic nucleus, and prokaryotic redox folds shared by bacteria and archaea. Together, they outline a layered, chimeric history in which eukaryotes inherited metabolic systems from bacteria and informational architectures from archaea. Lineage‐exclusive domains reflect divergent adaptive strategies: regulatory recombination in eukaryotes, structural optimization in bacteria, and metabolic conservatism in archaea. The analysis indicates that the proteomic diversity of modern life emerges from reorganizing a small set of ancient, versatile architectures that continue to sustain biology today. Beyond evolutionary insight, this structure‐based framework may provide a useful basis for identifying robust and portable protein scaffolds, informing efforts in protein engineering, synthetic biology, and heterologous expression, where fold stability and cross‐lineage compatibility are critical considerations.

## 4 Methods

### 4.1 Data Sources and Preprocessing

All protein domain data were obtained from the ECOD database, which provides hierarchical annotations of protein structures and sequence‐based homologs. Each ECOD domain entry includes both structural classification (A → X → H → T → F levels: Architecture, Possible homology, Homology, Topology, and Family) and taxonomic information derived from the NCBI Taxonomy database. This study focused on the homology (H) level, which groups domains inferred to share a common evolutionary origin regardless of sequence divergence.

To control for redundancy and uneven sampling, all ECOD domains were clustered at 99% sequence identity using CD‐HIT, producing nonredundant domain clusters referred to here as f99 clusters. This step collapses nearly identical domain sequences (arising from strain variation, gene duplication, allelic variants, or repeated experimental sampling) into a single representative unit. The use of f99 clusters minimizes inflation of domain abundance caused by overrepresented sequences, particularly in well‐studied organisms and in experimentally determined PDB entries. Unless otherwise specified, all quantitative analyses in this study are based on f99 clusters. Each f99 cluster inherits all taxonomic labels associated with its constituent sequences. If a cluster contains members from multiple superkingdoms, all corresponding lineages are considered present for that H‐group. Viral domains were excluded from the analysis due to inconsistent taxonomy and incomplete proteome coverage in the ECOD dataset.

### 4.2 Selection of Model Organisms

Analyses were restricted to 44 model organisms with complete proteome coverage in ECOD (Figure 1). This selection includes 28 eukaryotes, 15 bacteria, and one archaeon; their full taxonomical labels are listed in Table S1.

### 4.3 Superkingdom Occupancy Analysis

Each H‐group was analyzed for its presence across the three superkingdoms: Eukaryota (E), Bacteria (B), and Archaea (A). An H‐group was considered present in a given organism or superkingdom if any cluster belonging to that H‐group contained at least one sequence labeled with the corresponding taxonomy ID. Based on occupancy, all H‐groups were classified into seven mutually exclusive categories of EBA, EB, EA, BA, E, B, and A.

### 4.4 Enrichment Analysis

To examine lineage bias among universal H‐groups, an enrichment score was calculated for each superkingdom. The background frequency was defined as the proportion of f99 clusters assigned to each superkingdom across all H‐groups (E = 0.9009, B = 0.0958, and A = 0.00339). For each individual H‐group, the observed frequency of each superkingdom was computed as the fraction of its constituent clusters belonging to that lineage. The enrichment score for a given superkingdom was then defined as:

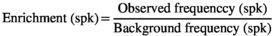




An enrichment value greater than 1 indicates that an H‐group is overrepresented in that superkingdom relative to the overall composition of the dataset.

### 4.5 Functional Annotation of Representative H‐Groups

Functional themes assigned to representative H‐groups were derived from well‐established literature describing canonical members of each structural family (e.g., P‐loop NTPases and Rossmann‐like dehydrogenases, TIM barrels, helix–turn–helix transcription factors). Because ECOD H‐groups are defined by structural homology rather than specific biochemical activity, individual members may perform multiple or context‐dependent functions. The functional categories used in this study represent predominant or historically characterized roles associated with each structural family and are intended for biological interpretation rather than formal functional enrichment analysis.

### 4.6 Use of Artificial Intelligence Tools

Large language model–based artificial intelligence tools (ChatGPT and OpenAI) were used to assist with language editing and improvement of manuscript clarity during preparation of the text. These tools were used only for drafting assistance, wording refinement, and grammatical editing. All scientific interpretations, data analyses, figures, and conclusions were developed and verified by the authors. No AI tools were used to generate research results, perform analyses, or produce scientific conclusions presented in this study.

## Author Contributions

**Nick V. Grishin:** funding acquisition, conceptualization, supervision. **Rui Guo:** conceptualization, methodology, investigation, writing – original draft, writing – review and editing, visualization, data curation, validation, formal analysis. **Jimin Pei:** writing – review and editing. **Jing Zhang:** data curation. **Qian Cong:** data curation, funding acquisition, supervision. **R. Dustin Schaeffer:** conceptualization, supervision, data curation, writing – review and editing, funding acquisition.

## Funding

This work was supported by the National Institute of Allergy and Infectious Diseases (1K99AI180984‐01A1), the National Institute of General Medical Sciences (GM147367 to R.D.S.; GM160468 to Q.C.), the National Science Foundation (MED230034, MED240004, and DBI 2224128 to N.V.G.), the Texas Advanced Computing Center (MCB24018 and MCB23014), and the Welch Foundation (I‐1505 to N.V.G.; I‐2095‐20220331 to Q.C.).

## Conflicts of Interest

The authors declare no conflicts of interest.

## Supporting information


**Table S1:** Taxonomical labels of 44 model organisms.
**Table S2:** Top 20 H‐groups absent from the 44 model organisms.
**Table S3:** 126 universal H‐groups existed in all 44 model organisms.
**Table S4:** Representatives of EB H‐groups.
**Table S5:** Representatives of EA H‐groups.
**Table S6:** BA H‐groups.
**Table S7:** Representatives of Eukaryota‐only H‐groups.
**Table S8:** Representatives of Bacteria‐only H‐groups.
**Table S9:** Archaea‐only H‐groups.
**Table S10:** Abbreviation of H group names used in the manuscript and plots.

## Data Availability

Data used in this analysis can be found at https://github.com/gr‐grey/ecod_domain_superkingdom.
