# Supplementary material for: Universal and Lineage‐Specific Patterns in the Distribution of ECOD Domain Homology Groups Across Superkingdoms
Source: Proteins. 2026 May 3;94(10):1644–57. doi: 10.1002/prot.70144 (PMC13539645; doi:10.1002/prot.70144)
Supplement: Supplementary file 1 [file PROT-94-1644-s001.docx]

**Supplementary Information**

## **Universal and Lineage-Specific Patterns in the Distribution of ECOD Domain Homology Groups Across Superkingdoms**

## Rui Guo^1^, Jimin Pei^3,4^, Jing Zhang^3,4^, Qian Cong^3,4^, Nick V. Grishin^1,2^, R. Dustin Schaeffer^1*^

1. Department of Biophysics, University of Texas Southwestern Medical Center, Dallas, TX, USA.

2. Department of Biochemistry, University of Texas Southwestern Medical Center, Dallas, TX, USA.

3. Eugene McDermott Center for Human Growth and Development, University of Texas Southwestern Medical Center, Dallas, TX, USA.

4. Harold C. Simmons Comprehensive Cancer Center, University of Texas Southwestern Medical Center, Dallas, TX, USA.

*Corresponding author: Richard.Schaeffer@UTSouthwestern.edu

**List of content**

-	Table S1: Taxonomical labels of 44 model organisms.

-	Table S2. Top 20 H-groups absent from the 44 model organisms.

-	Table S3. 126 universal H-groups existed in all 44 model organisms.

-	Table S4. Representatives of EB H-groups.

-	Table S5. Representatives of EA H-groups.

-	Table S6. BA H-groups.

-	Table S7. Representatives of Eukaryota-only H-groups.

-	Table S8. Representatives of Bacteria-only H-groups.

-	Table S9. Archaea-only H-groups.

-	Table S10. Abbreviation of H group names used in the manuscript and plots.

**Table S1: Taxonomical labels of 44 model organisms.**

| **Organism** | **f99 cluster count** | **Phylum** | **Kingdom** | **Superkingdom** |
| --- | --- | --- | --- | --- |
| *Homo sapiens* | 74790 | Chordata | Metazoa | Eukaryota |
| *Danio rerio* | 43084 | Chordata | Metazoa | Eukaryota |
| *Mus musculus* | 41370 | Chordata | Metazoa | Eukaryota |
| *Rattus norvegicus* | 15732 | Chordata | Metazoa | Eukaryota |
| *Caenorhabditis elegans* | 34853 | Nematoda | Metazoa | Eukaryota |
| *Trichuris trichiura* | 19891 | Nematoda | Metazoa | Eukaryota |
| *Strongyloides stercoralis* | 17885 | Nematoda | Metazoa | Eukaryota |
| *Onchocerca volvulus* | 14490 | Nematoda | Metazoa | Eukaryota |
| *Dracunculus medinensis* | 13603 | Nematoda | Metazoa | Eukaryota |
| *Wuchereria bancrofti* | 12533 | Nematoda | Metazoa | Eukaryota |
| *Brugia malayi* | 10687 | Nematoda | Metazoa | Eukaryota |
| *Drosophila melanogaster* | 28234 | Arthropoda | Metazoa | Eukaryota |
| *Schistosoma mansoni* | 26459 | Platyhelminthes | Metazoa | Eukaryota |
| *Glycine max* | 83771 | Streptophyta | Viridiplantae | Eukaryota |
| *Oryza sativa Japonica Group* | 48922 | Streptophyta | Viridiplantae | Eukaryota |
| *Zea mays* | 40503 | Streptophyta | Viridiplantae | Eukaryota |
| *Arabidopsis thaliana* | 35068 | Streptophyta | Viridiplantae | Eukaryota |
| *Sporothrix schenckii ATCC 58251* | 15771 | Ascomycota | Fungi | Eukaryota |
| *Histoplasma capsulatum G186AR* | 14277 | Ascomycota | Fungi | Eukaryota |
| *Saccharomyces cerevisiae S288C* | 13348 | Ascomycota | Fungi | Eukaryota |
| *Paracoccidioides lutzii Pb01* | 9766 | Ascomycota | Fungi | Eukaryota |
| *Candida albicans SC5314* | 8293 | Ascomycota | Fungi | Eukaryota |
| *Schizosaccharomyces pombe 972h-* | 7743 | Ascomycota | Fungi | Eukaryota |
| *Trypanosoma cruzi strain CL Brener* | 18842 | Euglenozoa | None | Eukaryota |
| *Leishmania infantum* | 10166 | Euglenozoa | None | Eukaryota |
| *Trypanosoma brucei brucei TREU927* | 10086 | Euglenozoa | None | Eukaryota |
| *Dictyostelium discoideum* | 26925 | Evosea | None | Eukaryota |
| *Plasmodium falciparum 3D7* | 7008 | Apicomplexa | Apicomplexa | Eukaryota |
| *Pseudomonas aeruginosa PAO1* | 9318 | Pseudomonadota | Pseudomonadati | Bacteria |
| *Escherichia coli K-12* | 8532 | Pseudomonadota | Pseudomonadati | Bacteria |
| *Klebsiella pneumoniae* | 8270 | Pseudomonadota | Pseudomonadati | Bacteria |
| *Salmonella enterica subsp. enterica serovar Typhimurium str. LT2* | 7061 | Pseudomonadota | Pseudomonadati | Bacteria |
| *Shigella dysenteriae Sd197* | 5278 | Pseudomonadota | Pseudomonadati | Bacteria |
| *Haemophilus influenzae Rd KW20* | 2914 | Pseudomonadota | Pseudomonadati | Bacteria |
| *Neisseria gonorrhoeae FA 1090* | 2703 | Pseudomonadota | Pseudomonadati | Bacteria |
| *Campylobacter jejuni subsp. jejuni NCTC 11168 = ATCC 700819* | 3013 | Campylobacterota | Pseudomonadati | Bacteria |
| *Helicobacter pylori 26695* | 2322 | Campylobacterota | Pseudomonadati | Bacteria |
| *Mycobacterium tuberculosis H37Rv* | 6333 | Actinomycetota | Bacillati | Bacteria |
| *Mycobacterium ulcerans str. Harvey* | 5892 | Actinomycetota | Bacillati | Bacteria |
| *Mycobacterium leprae TN* | 1966 | Actinomycetota | Bacillati | Bacteria |
| *Enterococcus faecium* | 4280 | Bacillota | Bacillati | Bacteria |
| *Staphylococcus aureus subsp. aureus NCTC 8325* | 3947 | Bacillota | Bacillati | Bacteria |
| *Streptococcus pneumoniae R6* | 3012 | Bacillota | Bacillati | Bacteria |
| *Methanocaldococcus jannaschii DSM 2661* | 2652 | Methanobacteriota | Methanobacteriati | Archaea |

Superkingdom, kingdom and phylum labels for the 44 model organisms. Taxonomical names adopted from NCBI taxonomy standard. 4 organisms (Trypanosoma cruzi strain CL Brener, Leishmania infantum, Trypanosoma brucei brucei TREU927, Dictyostelium discoideum) do not have a kingdom label.

By restricting the domain organism sources to the 44 model organisms, some H-groups in ECOD were missing from the analysis. Of all ECOD H-groups, 294 were absent from the 44-organism set. Most of them are small H groups with fewer than 10 sequences.

**Table S2. Top 20 H-groups absent from the 44 model organisms.**

| **H-group name** | **H-group ID** | **f99 cluster count** |
| --- | --- | --- |
| Subtilisin inhibitor | 841.1 | 21 |
| N-terminal domain of GerK3 germinant receptor | 7506.1 | 16 |
| Variable surface antigen VlsE | 624.1 | 15 |
| Phycoerythrin 545 alpha-subunits | 851.1 | 14 |
| Peridinin-chlorophyll protein | 569.1 | 13 |
| Helical domain in glucansucrase GTF180 | 3339.1 | 13 |
| Hantavirus structural glycoprotein Gn ectodomain subdomain III | 1080.1 | 11 |
| CytB endotoxin-like (Pfam 01338) | 858.1 | 11 |
| Photosynthetic reaction centre (cytochrome subunit) | 139.3 | 11 |
| a+b domain in platelet binding protein GspB | 6107.1 | 10 |
| Oxalate Biosynthetic Component A (ObcA) insertion domains | 3853.1 | 9 |
| CRISPR-Cas system second helical domain | 1149.1 | 9 |
| Magnetochrome domain | 3778.1 | 8 |
| Heme-binding protein HmuY-related | 3077.1 | 8 |
| Ig-like domain in putative lipoprotein BF3042-related proteins | 3233.1 | 7 |
| Trimeric autotransporter adhesin Trp ring domain | 716.1 | 7 |
| Immunodominant antigen Pgp3 N-terminal domain | 3700.1 | 7 |
| CRISPR-Cas system RNase C2c2 N-terminal domain | 3804.1 | 7 |
| DNA polymerase II large subunit DP2 helical domain | 101.45 | 6 |
| Cytochrome P460 | 3437.1 | 6 |

Top 20 of the 294 H-groups missing from the 44 model organisms, ranked by the number of unique sequences of each H-group.

**Table S3. 126 universal H-groups existed in all 44 model organisms.**

| **H-group name** | **f99 cluster count** | **H-group ID** |
| --- | --- | --- |
| Immunoglobulin-related | 39217 | 11.1 |
| HTH | 27928 | 101.1 |
| P-loop domains-related | 26390 | 2004.1 |
| Rossmann-related | 16379 | 2003.1 |
| Protein kinase/SAICAR synthase/ATP-grasp | 14006 | 206.1 |
| beta-propeller | 11608 | 5.1 |
| ARM repeat | 11043 | 109.4 |
| SH3 | 8937 | 4.1 |
| RIFT-related | 8782 | 1.1 |
| TIM barrels | 7866 | 2002.1 |
| Ubiquitin-related | 7286 | 221.1 |
| Ribonuclease H-like | 6881 | 2484.1 |
| Histone-related | 6716 | 148.1 |
| Nucleic acid-binding protein | 6437 | 2.1 |
| Major facilitator superfamily (MFS) general substrate transporter | 6425 | 5050.1 |
| Class I glutamine amidotransferase-like | 5685 | 2007.1 |
| HAD domain-related | 4735 | 2006.1 |
| Thioredoxin-like | 4535 | 2485.1 |
| UDP-Glycosyltransferase/glycogen phosphorylase | 4316 | 7512.1 |
| LIM domain-like | 4037 | 377.1 |
| Rubredoxin-related | 3979 | 375.1 |
| SAM/DNA-glycosylase | 3748 | 102.1 |
| Double-stranded beta-helix | 3593 | 10.12 |
| Cysteine proteinases | 3472 | 219.1 |
| Nucleotide-diphospho-sugar transferases | 3347 | 7516.1 |
| Flavoproteins/Phosphotyrosine protein phosphatases-like | 2655 | 2007.2 |
| Periplasmic binding protein-like II | 2483 | 7523.1 |
| RuvA-C, UBA, CRAL/TRIO-N, HBS1 | 2411 | 103.1 |
| HUP domains | 2351 | 2005.1 |
| PDZ domain | 2253 | 7.1 |
| Metalloproteases ("zincins") catalytic domain | 2200 | 2498.1 |
| KH-domains | 1882 | 327.11 |
| sensor domains | 1854 | 223.1 |
| Acyl-CoA N-acyltransferases (Nat) | 1736 | 213.1 |
| Sodium:neurotransmitter symporter family (SNF)-like | 1703 | 5051.1 |
| PLP-dependent transferases | 1684 | 7577.1 |
| C-terminal domain in some PLP-dependent transferases | 1617 | 3016.1 |
| ACT-like | 1558 | 304.8 |
| alpha/beta-Hammerhead/Barrel-sandwich hybrid | 1539 | 325.1 |
| Homodimeric domain of signal transducing histidine kinase | 1498 | 605.1 |
| Ribosomal protein S5 domain 2-like | 1468 | 212.1 |
| Adenylyl and guanylyl cyclase catalytic domain-like | 1432 | 304.48 |
| Ferritin/Heme oxygenase | 1316 | 150.1 |
| Metallo-dependent phosphatases | 1281 | 246.2 |
| Restriction endonuclease-like | 1185 | 2008.1 |
| The "swivelling" beta/beta/alpha domain | 1169 | 2487.1 |
| Type II ABC exporter transmembrane domain-related | 1003 | 1075.1 |
| Thiolase-like | 962 | 7581.1 |
| Class II aaRS and biotin synthetases | 954 | 314.1 |
| 6-phosphogluconate dehydrogenase C-terminal domain-like | 948 | 129.1 |
| Ntn | 943 | 210.1 |
| Zn-dependent exopeptidases | 933 | 2011.1 |
| Nucleotidyltransferase | 916 | 316.1 |
| ATPase domain of HSP90 chaperone/DNA topoisomerase II/histidine kinase | 909 | 225.1 |
| Thiamin diphosphate-binding fold (THDP-binding) | 909 | 7574.1 |
| FKBP-like | 894 | 284.1 |
| Calcium ATPase transmembrane domain-related | 854 | 5073.1 |
| Cytidine deaminase-like | 827 | 2492.1 |
| ClpP/crotonase | 815 | 2486.1 |
| Nudix | 774 | 221.4 |
| Drug/Metabolite transporter | 750 | 5059.1 |
| LuxS, MPP, ThrRS/AlaRS common domain | 698 | 309.1 |
| EF-G C-terminal domain-like | 687 | 304.24 |
| Terpenoid synthases | 668 | 141.1 |
| Metallo-hydrolase/oxidoreductase | 666 | 247.1 |
| Glyoxalase/Bleomycin resistance protein/Dihydroxybiphenyl dioxygenase | 644 | 211.1 |
| CBS-domain | 619 | 282.1 |
| Glyceraldehyde-3-phosphate dehydrogenase-like, C-terminal domain | 551 | 298.1 |
| PRTase-like | 538 | 7573.1 |
| FAD-linked reductases-C | 514 | 244.1 |
| Pseudouridine synthase | 462 | 304.102 |
| HD-domain/PDEase-like | 462 | 131.1 |
| Isocitrate/Isopropylmalate dehydrogenase-like | 457 | 7510.1 |
| Cation-proton antiporter (CPA) | 456 | 3236.1 |
| Trimeric LpxA-like enzymes | 442 | 208.1 |
| Uracil Transporter UraA | 434 | 3226.1 |
| Anticodon-binding domain of Class II aaRS | 429 | 7502.1 |
| Ribokinase-like | 427 | 2003.6 |
| alpha/beta knot | 425 | 2488.1 |
| Phosphoglucomutase, first 3 domains | 423 | 7528.1 |
| Aconitase iron-sulfur domain | 415 | 7542.1 |
| Anticodon-binding domain of a subclass of class I aminoacyl-tRNA synthetases | 411 | 140.1 |
| Ribosomal protein S2/SIS domain | 404 | 2007.6 |
| tRNA-binding arm | 398 | 192.7 |
| L30e-like | 395 | 301.1 |
| DNA polymerase III clamp loader subunits, C-terminal domain | 359 | 138.1 |
| HIT-related | 345 | 312.1 |
| Creatinase/aminopeptidase | 345 | 283.1 |
| 4Fe-4S ferredoxin | 343 | 205.1 |
| Band 7/SPFH domain | 323 | 230.5 |
| NAD kinase beta sandwich domain-like | 311 | 4004.1 |
| H2TH | 299 | 102.2 |
| HEPN | 296 | 601.7 |
| Peptidyl-tRNA hydrolase-like | 293 | 2011.2 |
| TK C-terminal domain-like | 282 | 7522.1 |
| L-aspartase C-terminal domain-like | 272 | 4953.1 |
| Glutamine synthetase/guanido kinase | 256 | 321.1 |
| FtsH protease domain-like | 230 | 4070.1 |
| L-aspartase N-terminal domain-like | 226 | 4952.1 |
| L-aspartase middle domain-like | 221 | 602.1 |
| STT3/PglB/AglB transmembrane domain | 207 | 3352.1 |
| Creatinase/prolidase N-terminal domain | 205 | 2484.3 |
| GHMP Kinase, C-terminal domain | 197 | 304.1 |
| S-adenosylmethionine synthetase | 195 | 327.17 |
| Probable ACP-binding domain of malonyl-CoA ACP transacylase | 186 | 304.11 |
| Ribosomal protein L10-like | 186 | 304.6 |
| Phosphoglucomutase, C-terminal domain | 174 | 331.2 |
| CDP-alcohol phosphotransferase helical bundle domain | 168 | 3883.1 |
| C-terminal domain in DHH phosphoesterases | 166 | 5104.1 |
| Nucleoside diphosphate kinases | 160 | 304.34 |
| LexA/Signal peptidase | 159 | 4.11 |
| Enolase N-terminal domain-like | 149 | 218.1 |
| Ribosomal protein L6 | 144 | 318.1 |
| Ribosomal proteins L15p and L18e | 141 | 2490.3 |
| a+b domain in Ribosomal protein L1 | 139 | 898.1 |
| Ribosomal protein S10 | 139 | 304.44 |
| ITPase-like | 135 | 7504.1 |
| Rossmann-like domain in Ribosomal protein L1 | 129 | 2007.25 |
| YrdC/RibB | 114 | 297.1 |
| GIY-YIG endonuclease | 94 | 821.1 |
| Ribosomal protein L11/L12e N-terminal domain | 89 | 3781.1 |
| Initiation factor IF2/eIF5b, domain 3 | 81 | 7526.1 |
| dUTPase-like | 77 | 70.2 |
| Uracil-DNA glycosylase-like | 77 | 7569.1 |
| Putative anticodon-binding domain of alanyl-tRNA synthetase (AlaRS) | 73 | 613.1 |
| Ribosomal protein S8, C-terminal domain | 69 | 4988.1 |

H-groups existed in all 44 organisms examined in this study, ranked by unique sequence count (clustered by 99% similarity).

**Table S4. Representatives of EB H-groups.**

| **H-group name** | **f99 cluster count** | **H-group ID** |
| --- | --- | --- |
| alpha/beta-Hydrolases | 4036 | 7579.1 |
| Chaperone J-domain | 1610 | 650.1 |
| Transmembrane heme-binding four-helical bundle | 1143 | 5069.1 |
| NTF2-like | 987 | 243.1 |
| helical hairpin of HlyD-like secretion proteins | 963 | 5086.1 |
| Rossmann-like domain in Acetyl-CoA synthetase-like proteins | 950 | 7584.1 |
| Type I ABC exporter transmembrane domain fold | 914 | 1075.4 |
| GroES-related | 824 | 236.1 |
| a+b domain in acetyl-CoA synthetase-like proteins | 818 | 327.5 |
| Thioesterase/thiol ester dehydrase-isomerase | 772 | 222.1 |
| DNase I-like | 739 | 246.3 |
| HSP40/DnaJ peptide-binding domain | 626 | 67.1 |
| Cyclophilin-like | 541 | 75.1 |
| Glycerol-3-phosphate (1)-acyltransferase | 439 | 7558.1 |
| Acyl-carrier protein (ACP) | 379 | 132.1 |
| Peptidyl-tRNA hydrolase domain-like | 286 | 330.3 |
| Ribonuclease PH domain 2 | 248 | 245.1 |
| Aminomethyltransferase folate-binding domain | 171 | 304.107 |
| Transmembrane Bax inhibitor motif (TMBIM) proteins | 147 | 3890.1 |
| alpha-helical domain in type II DNA topoisomerase | 132 | 4016.1 |
| Head domain of nucleotide exchange factor GrpE | 113 | 706.1 |
| L9 N-domain-like | 101 | 4076.1 |
| FtsH Periplasmic Domain | 86 | 4263.2 |
| a+b domain in type II DNA topoisomerase | 81 | 4014.1 |

24 Eukaryota + Bacteria H-groups existed in all 28 Eukaryota and 15 Bacteria organisms, ranked by unique sequence count (clustered by 99% similarity).

**Table S5. Representatives of EA H-groups.**

| **H-group name** | **f99 cluster count** | **H-group ID** |
| --- | --- | --- |
| Rossmann-like domain in prismane-like proteins | 15 | 7586.1 |
| GxGD membrane protease | 12 | 3457.1 |
| FdhD/NarQ beta-grasp fold domain | 11 | 221.1 |
| Antigen MPT63/MPB63 (immunoprotective extracellular protein) | 11 | 11.4 |
| Putative triphosphoribosyl-dephospho-coA synthase | 11 | 3076.1 |
| Nicotinate mononucleotide:5,6-dimethylbenzimidazole phosphoribosyltransferase (CobT) | 10 | 7553.1 |
| Fe-S cluster binding domain in carbon monoxide dehydrogenase corrinoid/iron-sulfur protein gamma subunit | 8 | 3858.1 |
| DisA nucleotide-binding domain | 6 | 4279.1 |
| Precorrin-8X methylmutase CbiC/CobH | 6 | 2007.7 |
| Aspartate carbamoyltransferase, Regulatory-chain, N-terminal domain | 6 | 304.38 |
| YfbU-like, N-terminal subdomain | 5 | 4359.1 |
| Bacterial fluorinating enzyme, N-terminal domain | 5 | 7540.1 |
| CofE-like | 5 | 4093.1 |
| CofE insertion domain | 5 | 101.25 |
| CobE/CbiG C-terminal domain-like | 5 | 4262.1 |
| Nitrogenase accessory factor-like | 5 | 2484.4 |
| Rossmann-like domain in CbiD | 4 | 7582.1 |
| CbiG linker domain | 3 | 4975.1 |
| NucS N-terminal domain | 3 | 220.5 |
| STT3/PglB/AglB beta-barrel domain | 3 | 3111.1 |
| Fe-binding domain in prismane-like proteins | 3 | 4017.1 |
| Aldehyde ferredoxin oxidoreductase-C | 2 | 169.1 |
| Aldehyde ferredoxin oxidoreductase, N-terminal domain | 2 | 806.1 |

23 Eukaryota + Archaea H-groups existed in all 28 Eukaryota and 1 Archaea organisms, ranked by unique sequence count (clustered by 99% similarity).

**Table S6. BA H-groups.**

| **H-group name** | **Bacteria**  **organisms** | **Archaea**  **organisms** | **f99 cluster count** | **H-group ID** |
| --- | --- | --- | --- | --- |
| FdhD/NarQ beta-grasp fold domain | 10 | 1 | 11 | 221.1 |
| GxGD membrane protease | 9 | 1 | 12 | 3457.1 |
| Fe-S cluster binding domain in carbon monoxide dehydrogenase corrinoid/iron-sulfur protein gamma subunit | 7 | 1 | 8 | 3858.1 |
| Nicotinate mononucleotide:5,6-dimethylbenzimidazole phosphoribosyltransferase (CobT) | 7 | 1 | 10 | 7553.1 |
| Putative triphosphoribosyl-dephospho-coA synthase | 6 | 1 | 11 | 3076.1 |
| Precorrin-8X methylmutase CbiC/CobH | 5 | 1 | 6 | 2007.7 |
| DisA nucleotide-binding domain | 5 | 1 | 6 | 4279.1 |
| Aspartate carbamoyltransferase, Regulatory-chain, N-terminal domain | 5 | 1 | 6 | 304.38 |
| Rossmann-like domain in prismane-like proteins | 4 | 1 | 15 | 7586.1 |
| YfbU-like, N-terminal subdomain | 4 | 1 | 5 | 4359.1 |
| Bacterial fluorinating enzyme, N-terminal domain | 4 | 1 | 5 | 7540.1 |
| Antigen MPT63/MPB63 (immunoprotective extracellular protein) | 4 | 1 | 11 | 11.4 |
| CbiG linker domain | 3 | 1 | 3 | 4975.1 |
| CofE-like | 3 | 1 | 5 | 4093.1 |
| CofE insertion domain | 3 | 1 | 5 | 101.25 |
| Rossmann-like domain in CbiD | 3 | 1 | 4 | 7582.1 |
| CobE/CbiG C-terminal domain-like | 3 | 1 | 5 | 4262.1 |
| NucS N-terminal domain | 2 | 1 | 3 | 220.5 |
| STT3/PglB/AglB beta-barrel domain | 2 | 1 | 3 | 3111.1 |
| Nitrogenase accessory factor-like | 1 | 1 | 5 | 2484.4 |
| Aldehyde ferredoxin oxidoreductase-C | 1 | 1 | 2 | 169.1 |
| Fe-binding domain in prismane-like proteins | 1 | 1 | 3 | 4017.1 |
| Aldehyde ferredoxin oxidoreductase, N-terminal domain | 1 | 1 | 2 | 806.1 |

23 Bacteria + Archaea H-groups ranked by the number of bacteria organisms each H-group.

**Table S7. Representatives of Eukaryota-only H-groups.**

| **H-group name** | **f99 cluster count** | **H-group ID** |
| --- | --- | --- |
| POZ domain | 2080 | 226.1 |
| F-box domain | 1633 | 145.1 |
| Calponin-homology domain-like | 1384 | 193.1 |
| Mitochondrial ADP/ATP carrier | 1085 | 5076.1 |
| CCCH zinc finger | 957 | 906.1 |
| GTPase activation domain, GAP | 782 | 189.1 |
| Gelsolin-like | 760 | 224.1 |
| TRAF domain-like | 698 | 11.1 |
| HMG-box | 587 | 190.1 |
| Acyl-CoA binding protein-like | 571 | 197.1 |
| PX domain | 446 | 277.1 |
| triple barrel | 411 | 59.1 |
| Zinc hairpin stack | 396 | 4106.1 |
| Rossmann-like domains in Sec1/munc18-like proteins | 316 | 7585.1 |
| DLC | 296 | 864.1 |
| Formin homology 2 domain (FH2 domain) | 279 | 626.1 |
| Hect, E3 ligase catalytic domain | 244 | 261.1 |
| Palmitoyltransferase DHHC transmembrane domain | 235 | 7015.1 |
| PAH2 domain | 235 | 509.1 |
| C-terminal tetramerization domain of Utp1/Utp21/Utp12/Utp13 | 226 | 109.54 |
| Conserved domain common to transcription factors TFIIS, elongin A, CRSP70 | 224 | 611.3 |
| TMEM16 lipid scramblase transmembrane domain | 219 | 3914.1 |
| Helical domain in TOPLESS related protein 2 (TPR2) | 217 | 109.46 |
| HIT/MYND zinc finger-like | 216 | 377.9 |
| Surp module (SWAP domain) | 197 | 574.1 |
| 26S proteasome regulatory subunit RPN8/RPN11 C-terminal domain | 182 | 3748.1 |
| a+b domain in cullin-like proteins | 170 | 4001.1 |
| alpha-helical domain in sec1/munc18-like (SM) proteins | 156 | 4015.1 |
| WWE domain | 150 | 4357.1 |
| Subdomain of clathrin and coatomer appendage domain | 146 | 331.9 |
| VPS9 domain | 136 | 627.1 |
| Nucleoporin NUP85/Nucleoporin NUP145 C-terminal domain | 133 | 109.21 |
| Phosphoinositide phosphatase SAC1 N-terminal domain | 133 | 3257.1 |
| PABC(PABP) domain | 129 | 143.1 |
| Differentially expressed in normal cells and neoplasia (DENN) domain | 121 | 2004.5 |
| TLDC domain of oxidation resistance protein 2 | 115 | 9.15 |
| Bromodomain-containing protein 4 ET domain | 96 | 3525.1 |
| Arp2/3 complex subunits | 90 | 241.6 |
| Zinc finger domain of DNA polymerase-alpha | 90 | 375.1 |
| E2-binding domain of E1 | 84 | 221.7 |
| FAD-dependent thiol oxidase | 83 | 601.21 |
| 40S ribosomal protein S7-A | 81 | 327.18 |
| Acidic mitochondrial matrix protein p32 | 80 | 897.1 |
| 26S proteasome regulatory subunit RPN7 C-terminal helix | 75 | 3749.4 |
| RuBisCo LSMT C-terminal, substrate-binding domain | 72 | 630.1 |
| Proteasome/cyclosome (PC) repeat | 66 | 109.35 |
| RPL28 | 64 | 3209.1 |
| Alix V domain | 63 | 3285.1 |
| 60S ribosomal protein rpL36 (L36e) | 61 | 3166.1 |
| Zuotin homology domain (ZHD) | 59 | 1108.1 |
| RABEX-5 helical domain | 57 | 633.24 |
| Functional domain of the splicing factor Prp18 | 55 | 544.1 |
| Pre-mRNA-splicing factor 38A | 51 | 109.5 |
| Eukaryotic RPB5 N-terminal domain | 47 | 2008.3 |
| RPL13 | 46 | 3210.1 |
| Nuclease A inhibitor (NuiA)-related | 41 | 243.9 |
| eEF1-gamma domain | 38 | 304.46 |
| ERO1-like | 37 | 526.1 |
| Fe-only hydrogenase | 37 | 7556.1 |
| Protein CLP1 jelly-roll domain | 36 | 10.34 |
| MAL13P1.257-like | 36 | 4135.1 |
| ubiquitin-binding domain from PLAA | 34 | 3075.1 |
| Pre-mRNA-splicing factor BUD31 | 30 | 3952.1 |

63 Eukaryotic exclusive H-group that are found in all 28 Eukaryotic organisms, ranked by unique sequence count (clustered by 99% similarity).

**Table S8. Representatives of Bacteria-only H-groups.**

| **h_group_name** | **Bacterial organisms** | **f99 cluster count** | **h_group_id** |
| --- | --- | --- | --- |
| DNA-binding domain in UvrA | 15 | 17 | 3326.1 |
| UvrB-binding domain of UvrA | 15 | 14 | 3325.1 |
| Lmo1499 protein N-terminal domains | 13 | 27 | 3536.1 |
| a/b domain in QueA-like proteins (Pfam 02547) | 12 | 12 | 4013.1 |
| OmpA-like | 11 | 54 | 301.3 |
| Phospholipid translocation channel MlaA | 8 | 10 | 3466.1 |
| YgfB-like | 7 | 10 | 635.1 |
| Bacterial RNA polymerase subunit epsilon | 7 | 9 | 3115.6 |
| C-terminal domain of DNA Polymerase III subunit tau | 7 | 7 | 327.14 |
| Cell-division inhibitor MinC-C | 7 | 6 | 207.5 |
| Penicillin-binding protein 2x (pbp-2x)-C | 6 | 33 | 294.2 |
| PTS IIb component | 6 | 21 | 7537.1 |
| Flagellar hook protein flgE D1 domain | 6 | 18 | 4167.1 |
| Baseplate wedge protein gp6 helical domain | 6 | 10 | 1049.2 |
| RNA polymerase sigma-54 factor N-terminal region | 6 | 10 | 1004.1 |
| Domain 1 of outer membrane lipoprotein Wza | 6 | 7 | 6016.1 |
| Tetrahydrodipicolinate-N-succinlytransferase, N-terminal 3-helical domain | 6 | 6 | 3001.1 |
| Flagellar cap protein FliD helical bundle domain | 6 | 6 | 601.5 |
| Tetrahydrodipicolinate N-succinyletransferase N-terminal domain | 6 | 6 | 3610.1 |
| Barrel domain in flagellar protein FlgA | 6 | 5 | 3174.2 |
| CheW | 5 | 25 | 2.1 |
| 4Fe-4S protein WhiB1 | 5 | 14 | 1022.2 |
| Regulator of polyketide synthase expression N-terminal domain | 5 | 11 | 304.154 |
| L,D-transpeptidase C-terminal domain | 5 | 10 | 3743.1 |
| Glycyl radical domain | 5 | 10 | 7090.1 |
| Uncharacterized protein VC_A0919 | 5 | 9 | 3313.1 |
| Outer membrane protein assembly factor BamC | 5 | 8 | 331.22 |
| all-beta domain in nickel-iron hydrogenase large subunit | 5 | 8 | 4046.1 |
| Tsx-like channel | 5 | 7 | 5084.6 |
| Helical insertion of YcjX stress protein | 5 | 5 | 7110.1 |
| YehR-like | 5 | 5 | 4142.1 |
| Regulatory domain of isocitrate dehydrogenase kinase/phosphatase | 5 | 5 | 3222.1 |
| lantibiotic self-resistance lipoprotein MlbQ | 5 | 5 | 3982.1 |
| Dimerization domain of erythronate-4-phosphate dehydrogenase PdxB | 5 | 5 | 6096.1 |
| Ribosomal protein S12 methylthiotransferase accessory factor YcaO C-terminal domain | 5 | 5 | 109.41 |
| Urease metallochperone UreE-N | 5 | 5 | 55.1 |
| Uncharacterized protein VC_0899 | 5 | 4 | 3623.1 |
| Lytic transglycosylase MltC N-terminal domain | 5 | 4 | 312.2 |

Showing H-groups with bacterial organism count >= 5. There are 38 H-groups, ranked by organisms count.

**Table S9. Archaea-only H-groups.**

| **H-group name** | **f99 cluster count** | **H-group ID** |
| --- | --- | --- |
| O-phosphoseryl-tRNA synthetase C-terminal domain | 2 | 304.135 |
| B12 binding domain in carbon monoxide dehydrogenase corrinoid/iron-sulfur protein gamma subunit | 2 | 7595.1 |
| first a+b domain in acetyl-CoA synthase | 2 | 4223.1 |
| second a+b domain in acetyl-CoA synthase | 2 | 4971.1 |
| Outer membrane-associated lipoprotein TP0453 | 2 | 881.3 |
| Formylmethanofuran:tetrahydromethanopterin formyltransferase | 2 | 304.21 |
| Pyruvoyl-dependent histidine and arginine decarboxylases | 1 | 303.1 |
| AF1531-like | 1 | 236.3 |
| Dhaf4260 N-terminal domain | 1 | 218.4 |
| Tetrahydromethanopterin S-methyltransferase subunit A | 1 | 2007.2 |
| Endonuclease PI-TkoII domain IV | 1 | 1057.1 |
| DNA polymerase D large subunit DP2 N-terminal domain | 1 | 3349.1 |
| gp10 domain 2 and domain 3 | 1 | 3386.2 |
| Cascade subunit Csa5 | 1 | 3572.1 |
| primase chain A, C-terminal domain | 1 | 4076.4 |
| SSHS domain in O-phosphoseryl-tRNA synthetase | 1 | 4012.2 |
| GatD N-terminal domain-like | 1 | 4.17 |
| TrmB C-terminal domain-like | 1 | 4.15 |
| MMP1218-like | 1 | 6073.1 |
| N-terminal domain in DUF2118 family | 1 | 6047.1 |
| MJ1004, C-terminal domain | 1 | 7103.1 |
| Methenyltetrahydromethanopterin cyclohydrolase | 1 | 869.1 |

22 H-groups in total, ranked by unique sequence count (clustered by 99% similarity).

**Table S10. Abbreviation of H group names used in the manuscript and plots.**

| **H-group ID** | **H-group name** | **short name** |
| --- | --- | --- |
| 1.1 | RIFT-related | RIFT |
| 10.1 | Concanavalin A-like | Concvln |
| 10.12 | Double-stranded beta-helix | dsBhlx |
| 10.2 | Nucleoplasmin-like/VP (viral coat and capsid proteins) | ViralCoat |
| 10.32 | Galactose-binding domain-like | Galactose |
| 101.1 | HTH | HTH |
| 102.1 | SAM/DNA-glycosylase | SAM/Glyco |
| 102.2 | H2TH | H2TH |
| 103.1 | RuvA-C, UBA, CRAL/TRIO-N, HBS1 | RuvA/UBA/CRALN |
| 108.1 | EF-hand-related | EF-hnd |
| 109.3 | Ankyrin repeat | Ankyrin |
| 109.4 | ARM repeat | ARM |
| 11.1 | Immunoglobulin-related | Igl |
| 11.2 | C2 domain | C2 |
| 148.1 | Histone-related | Histone |
| 149.1 | Cytochrome P450 | P450 |
| 167.1 | Ribosomal protein S7 | Ribo S7 |
| 192.29 | bMERB domain (bivalent Mical/EHBP Rab binding) | bMERB |
| 192.4 | Ribosomal protein L29 (L29p) | Ribo L29 |
| 192.7 | tRNA-binding arm | tRNA arm |
| 192.8 | Eukaryotic DNA topoisomerase I, dispensable insert domain | Topoisomerase |
| 2.1 | Nucleic acid-binding protein | NBD |
| 2002.1 | TIM barrels | TIM |
| 2003.1 | Rossmann-related | Rossman |
| 2004.1 | P-loop domains-related | Ploop |
| 2005.1 | HUP domains | HUP |
| 2006.1 | HAD domain-related | HAD |
| 2007.1 | Class I glutamine amidotransferase-like | C1-Gdt |
| 2007.6 | Ribosomal protein S2/SIS domain | S2/SIS |
| 2008.1 | Restriction endonuclease-like | Rstn Endo |
| 2011.2 | Peptidyl-tRNA hydrolase-like | PTH |
| 205.1 | 4Fe-4S ferredoxin | 4Fe-4S Fd |
| 206.1 | Protein kinase/SAICAR synthase/ATP-grasp | PK |
| 207.1 | Leucine-rich repeats | LRR |
| 210.1 | Ntn | Ntn |
| 211.1 | Glyoxalase/Bleomycin resistance protein/Dihydroxybiphenyl dioxygenase | GBD |
| 212.1 | Ribosomal protein S5 domain 2-like | Ribo S5 |
| 213.1 | Acyl-CoA N-acyltransferases (Nat) | CoA Nat |
| 218.1 | Enolase N-terminal domain-like | Enolase-N |
| 218.2 | Ribosomal protein L22 | Ribo L22 |
| 219.1 | Cysteine proteinases | CysProt |
| 220.1 | PH domain-like | PH |
| 221.1 | Ubiquitin-related | Ubq |
| 223.1 | sensor domains | Sensor |
| 225.1 | ATPase domain of HSP90 chaperone/DNA topoisomerase II/histidine kinase | Hsp90/TopoII/HK |
| 230.2 | Ribosomal protein S3-C | Ribo S3-C |
| 247.1 | Metallo-hydrolase/oxidoreductase | Metallo-hydrolase |
| 2484.1 | Ribonuclease H-like | RnaseH |
| 2485.1 | Thioredoxin-like | Thiordxn |
| 2487.1 | The "swivelling" beta/beta/alpha domain | Swivel BBA |
| 2488.1 | alpha/beta knot | a/b-knot |
| 2490.3 | Ribosomal proteins L15p and L18e | Ribo L15p+ |
| 2498.1 | Metalloproteases ("zincins") catalytic domain | Zincin |
| 2498.5 | GroEL-intermediate domain like | GroEL-int |
| 282.1 | CBS-domain | CBS |
| 297.1 | YrdC/RibB | YrdC/RibB |
| 298.1 | Glyceraldehyde-3-phosphate dehydrogenase-like, C-terminal domain | GAPDH-C |
| 3010.1 | C-terminal subdomain in Lon-related proteases catalytic domains | LonP Ctd |
| 3016.1 | C-terminal domain in some PLP-dependent transferases | PLPctd |
| 304.1 | GHMP Kinase, C-terminal domain | GHMPK-C |
| 304.109 | Ribosomal proteins S24e, L23 and L15e | Ribo S24e+ |
| 304.11 | Probable ACP-binding domain of malonyl-CoA ACP transacylase | MAT-ACPbd |
| 304.24 | EF-G C-terminal domain-like | EF-G Ctd |
| 304.34 | Nucleoside diphosphate kinases | NDK |
| 304.44 | Ribosomal protein S10 | Ribo S10 |
| 304.9 | RNA-binding domain, RBD | RBD |
| 314.1 | Class II aaRS and biotin synthetases | C2-aaRS/BioS |
| 3174.1 | Ribosomal protein L14-related | Ribo L14 |
| 318.1 | Ribosomal protein L6 | L6 |
| 320.2 | Ribosomal protein S8, N-terminal domain | Ribo S8 Ntd |
| 321.1 | Glutamine synthetase/guanido kinase | GS/GK |
| 323.1 | CoA-dependent acyltransferases | CoA At |
| 325.1 | alpha/beta-Hammerhead/Barrel-sandwich hybrid | a/b-HH/BS |
| 327.11 | KH-domains | KH |
| 327.17 | S-adenosylmethionine synthetase | MAT |
| 328.2 | C-terminal domain of ProRS | ProRS Ctd |
| 375.1 | Rubredoxin-related | Rubr |
| 3755.3 | CT398 helical hairpin | CT398 |
| 376.1 | RING/U-box-like | RING |
| 377.1 | LIM domain-like | LIM |
| 3781.1 | Ribosomal protein L11/L12e N-terminal domain | Ribo L11 Ntd |
| 386.1 | beta-beta-alpha zinc fingers | BBA Zn |
| 3860.1 | Myosin VI lever arm | Myosin VI |
| 389.1 | EGF-related | EGF |
| 3939.1 | XRCC4 C-terminal oligomerization domain | XRCC4 |
| 4.1 | SH3 | SH3 |
| 4.11 | LexA/Signal peptidase | LexA/SignalPep |
| 4232.1 | Ribosomal protein bL28-related | Ribo bL28 |
| 4954.1 | central helical domain in RNA-polymerase beta-prime subunit | RNAP Hel |
| 4988.1 | Ribosomal protein S8, C-terminal domain | Ribo S8 Ctd |
| 5.1 | beta-propeller | Bprop |
| 5001.1 | Family A G protein-coupled receptor-like | GPCR |
| 5050.1 | Major facilitator superfamily (MFS) general substrate transporter | MFS |
| 5073.1 | Calcium ATPase transmembrane domain-related | Ca-ATPase TM |
| 5104.1 | C-terminal domain in DHH phosphoesterases | DHH-C |
| 511.1 | Heat shock protein 70kD (HSP70), peptide-binding domain | HSP70 PBD |
| 601.7 | HEPN | HEPN |
| 616.1 | S15/NS1 RNA-binding domain | S15/NS1 RBD |
| 632.7 | Heat shock protein 70kD (HSP70), C-terminal subdomain | HSP70 Ctd |
| 7.1 | PDZ domain | PDZ |
| 70.2 | dUTPase-like | dUTPase |
| 7502.1 | Anticodon-binding domain of Class II aaRS | C2-aaRS ABD |
| 7504.1 | ITPase-like | ITPase |
| 7510.1 | Isocitrate/Isopropylmalate dehydrogenase-like | IDH/IPMDH |
| 7512.1 | UDP-Glycosyltransferase/glycogen phosphorylase | UDP phsp |
| 7516.1 | Nucleotide-diphospho-sugar transferases | NDST |
| 7523.1 | Periplasmic binding protein-like II | PBP-II |
| 7524.1 | ALDH-like | ALDH |
| 7532.1 | Phosphoglycerate kinase domain 1 | PGK |
| 7541.1 | Molybdenum cofactor biosynthesis proteins | Moco Bio |
| 7542.1 | Aconitase iron-sulfur domain | Acn Fe-S |
| 7569.1 | Uracil-DNA glycosylase-like | UNG |
| 7574.1 | Thiamin diphosphate-binding fold (THDP-binding) | ThDP-bind |
| 7577.1 | PLP-dependent transferases | PLP |
| 7579.1 | alpha/beta-Hydrolases | ab Hyd |
| 7581.1 | Thiolase-like | Thiolase |
| 8002.1 | Zn-binding domain in glutaminyl-tRNA synthetase | GlnRS Zn |
| 838.1 | Ribosomal protein S19 | Ribo S19 |

H-groups full name in ECOD database and their short names used in the main manuscript and plots.
